# A review of smartphone applications designed to improve occupational health, safety, and well-being at workplaces

**DOI:** 10.1186/s12889-022-13821-6

**Published:** 2022-08-10

**Authors:** Iben Louise Karlsen, Peter Aske Svendsen, Johan Simonsen Abildgaard

**Affiliations:** 1 The National Research Centre for the Working Environment, Lersø Parkalle 105, 2100 Copenhagen, Denmark; 2 Department of Communication and Psychology, Aalborg University, A. C. Meyers Vænge 15, 2450 Copenhagen, Denmark; 3 Department of Organization, Copenhagen Business School, Kilevej 14A, 2000 Frederiksberg, Denmark

**Keywords:** Apps, Smartphone applications, Occupational health, Well-being, Technology, Digital health, e-health, m-health

## Abstract

**Background:**

As smartphones become more widespread, software applications for occupational health, safety and well-being (OHS&W) at work are increasing. There is sparse knowledge about the available apps and the research evidence of their effects. This study aims to identify available smartphone applications designed to improve OHS&W at workplaces, and examine to what extent the apps are scientifically validated.

**Methods:**

We searched the Danish App Store and Google Play for free OHS&W apps. Apps were included if they targeted OHS&W and were designed for workplace use. After categorizing the apps, we searched bibliographic databases to identify scientific studies on the ‘intervention apps’.

**Results:**

Altogether, 57 apps were included in the study; 19 apps were categorized as digital sources of information, 37 apps contained an intervention designed for workplace changes, and one app had too sparse information to be classified. Based on the publicly available information about the 37 intervention apps, only 13 had references to research. The bibliographic database search returned 531 publications, resulting in four relevant studies referring to four apps aimed at ergonomic measures, noise exposure, and well-being, which showed either limited effect or methodological limitations.

**Conclusion:**

There is no conceptual clarity about what can be categorized as an OHS&W app. Although some of the apps were developed based on scientific research, there is a need to evaluate the apps’ effects in promoting OHS&W. The sparse documentation of evidence should be kept in mind when applying apps to improve OHS&W.

**Supplementary Information:**

The online version contains supplementary material available at 10.1186/s12889-022-13821-6.

## Background

The proliferation of smartphones has led to a growth in the market for smartphone applications, commonly referred to as “apps”. With the increase in smartphone capabilities and device ownership, new possibilities and forms of use have emerged. Among other places, apps have entered the workplace as a new category of tools to improve occupational health, safety, and well-being at work (OHS&W). However, the new apps seem promising by being more sophisticated and more accessible to implement than classic health and safety tools, as a new addition to the health and safety toolbox, our knowledge of apps is still somewhat limited. The current study aims to review the available apps and present an overview of what exists to clarify the current status of OSH&W apps and what challenges lie ahead.

Digital technologies have dramatically changed working life in many ways in recent decades, including how to improve occupational health [1]. Not long ago, monitoring the work environment required a paper form handed out at the workplace or sent by mail, followed by a laborious process of collecting and comparing responses. Today, it is possible to measure employee satisfaction as often as desired, to send any number of work environment tools and guidelines directly into the target group's pockets through smartphone apps. Not only are processes accelerated, but also it is easier than ever to share ideas, retrieve information, and communicate with colleagues and managers, wherever and whenever desired. The market for OHS&W apps is growing rapidly and providing a wealth of approaches and opportunities for work environment professionals. However, with the emerging market of apps, there is sparse knowledge about the apps available.

An already large market for software applications is the healthcare field. Though apps are reasonably new in OSH&W, they have been used and researched more extensively in healthcare settings. Here, e-health (electronic health) and m-health (mobile health) have been gaining ground with expectations that the technology can help the process of enabling people to increase control over, and improve their health [2], making healthcare more accessible, and personal [3, 4].

Some of the potentials of e-health highlighted also apply to apps used for improving OHS&W. They can reach many individuals, are 24-h accessible, content can be updated at any time, and training can be repeated as often as desired [5]. Another advantage is that apps can activate and utilize the features of the smartphone, such as; notifications, sensors, GPS, audio/video recordings, camera, and access to the internet to provide instant feedback or support [6]. Conversely, there are possible negative implications of using smartphones to manage the OHS&W. Potential issues include; lack of transparency [7], concerns about data security, concerns about the smartphone or app being a tool for surveillance of the workers [8], the constant attention drawn to technology being a stressor [9], possible misinterpretations of the instant feedback on the screen [5], blurring of boundaries’ between paid work and personal life, and increased availability [10].

The pros and cons of apps likely also apply when it comes to OHS&W. In addition, when it comes to the fields of OSH&W, the potential risk of using apps is that they contribute to the framing of OHS&W as an individual rather than an organizational effort. Studies show how digital apps can promote particular visions of concepts like well-being [11]. In the case described by Islam et al. [11], the vision of ‘well-being’ promoted by the app under study was either one of individual freedom or collectivity – but not both [11]. For e-health within OHS&W, this is relevant concerning individualizing OHS&W work, as the arena for dealing with OHS&W risks shifting towards individual employees' smartphones at the expense of collective fora of the workplace [12]. This could lead to a reduced focus on organizational initiatives, which are generally accepted as important criteria for realizing improvements in OHS&W [13, 14]. Finally, a significant disadvantage is the lack of documented effects of the available apps.

Despite these advantages and disadvantages, the market of app-based tools for OHS&W is growing, and it is relevant to examine the field scientifically. So far, little scientific research has been done on apps in health promotion, and there is even less research about the use of apps as tools for interventions in the organizational context [5].

As a result, studies about apps in the field of Mental Health often evaluate which content is most popular among users and to which degree content is in line with evidence-based guidelines [4, 15, 16]. Reviews into the content of Mental Health Apps have found that most do not include key clinical focus points or are not in line with practice guidelines [16, 17]. The field of health promotion apps is thus characterized by a low evidence base [18]. Notable exceptions in the OHS&W field include a study by Bech et al. [19], who evaluated an app-based workplace intervention that provides psychological intervention based on the app-user answering the WHO-5 Scale [20] biweekly in the app (Howdy). This pilot study found indications of a positive effect of the app, specifically a shorter than expected time to return to average wellbeing. However, more studies are needed to confirm the findings.

Similarly, Sandal et al. [21] evaluated the effectiveness of an individually tailored self-management system delivered through an artificial intelligence-based app for pain-related disability in adults with low back pain. They found promising results in an RCT study involving 461 participants. However, the effect was too small to be clinically meaningful. Other studies have assessed the effect of OHS&W apps but as a pilot study or using preliminary designs [19, 22]. These studies point to the possible benefits of using apps in the OHS&W contexts, although, for now, there are sparse studies and a lack of clear effects. The research into the effects of OHS&W apps is growing, but it is a field still emerging and needs further research.

Research is needed into how apps are used, and their positive effects and potential pitfalls to create an overview of the effects of OHS&W apps. However, before such research can be fully utilized, a scientifical debate is needed to establish the exact definitions and characteristics of an OSH&W “app”, as there is not currently a stringent definition of this within OHS&W. As such, the present study works with a broad definition of an app as an application accessible through and designed for use on smartphones. Based on this definition, an app may also be accessed using a web browser. In the study, an OHS&W app is defined as occupational health, safety, and well-being tool accessible through smartphones.

The study aims to provide much-needed clarity on the field of OHS&W apps. We approach the app market from the same initial position as OHS&W professionals (i.e., looking at what is available in app stores). We do this instead of focusing exclusively on the few and not widely used apps that form the bulk of meta-analyses focusing on research evidence in Metal Health Apps [15, 23].

Despite the apparent growing prevalence and increased use of apps in OHS&W work, there is a lack of knowledge about the effect of using apps in OHS&W. There are few guidelines for selecting and using app-based interventions. The current study provides a review and content analysis of freely available OHS&W apps and assesses the number of studies that have been evaluated in scientific research. This study asks the following research questions:

- 1) Which apps are available for OHS&W?
- 2) Which organizations are bringing them to the market?
- 3) Which areas of OHS&W and level of the organization do the available apps target?
- 4) To what extent are the OHS&W apps scientifically validated? Are they based on scientific research? And are they scientifically validated?

## Method

### Data collection procedure

There has been sparse research into apps for OHS&W, though apps for mental health and well-being have been studied [15–17]. In order to gain an overview, the first step was to develop a search strategy. We mapped OHS&W apps by searching the two major app outlets, Apple’s App Store and Google’s Google Play. We used the following keywords in both databases: work environment, occupational health, occupation, work environment authority, productivity, leader, safety representative, union representative, sick leave, safety, lift, pain, well-being, stress, and mental health. The search terms were selected based on in-depth knowledge of the Danish field of occupational health.

We used two smartphones (one iPhone running IOS and one OnePlus running Android operating system) for the search. The two smartphones used had access to apps available through the Danish versions of the App Store and Google Play. The access we had through our Danish phones was limited to the apps available via the Danish version of the App Store and Google Play. Most likely, some apps were excluded due to this. Additionally, we searched in Google Play Store using a browser, a possibility that Apple’s App Store does not support. To search in Apple’s App Store using a browser, we used the homepage https://fnd.io/, a service that allowed us to adjust which national market edition of the App Store we wished to search. The search terms were in Danish; however, apps in English were also included if they appeared based on the search terms.

In the App Store and Google Play Store, it is not possible to set up search criteria and run the search as one would do in bibliographic databases; therefore, each search term was searched individually on App Store and Google Play Store. The apps suggested based on the search (“you might also like” section) were included in the study, if relevant. Each keyword served as a starting point for what resembled a snowball data collection method [24]. This resulted in several cross-references for each keyword; therefore, it is not telling to make a table showing how many apps we found on each keyword. The approach also made it necessary to make the first selection process part of the data collection phase. Hence, the first of two rounds of the data selection process took place while extracting the data. We assessed the apps based on the following criteria:

• Were the apps aimed at working life, workplaces, and occupational health?
• Did they concern an operationalization of occupational health topics?

The criteria meant that, for instance, coloring apps (coloring books for adults) found by the keyword search: stress were not included, as they were not aimed particularly at reducing stress at work. The second criteria meant that, for instance, apps on the correct usage of ladders were included as researchers deemed it an operationalization of an OSH&W topic, specifically safety.

This study is based only on the apps available through the App Store and Google Play Store. The present study does not include apps not available via these two channels, such as those specifically developed for or by a specific company for internal use. We conducted searches on the internet using the names of the apps. The identified data material about each app (webpages, articles, reviews, detailed descriptions of the application, etc.) was saved using the Ncapture web tool. We kept all the data material in Nvivo11, enabling us to code the data material.

In addition to the search on the App Store and Google Play Store, we searched InfoMedia, a database containing all Danish newspaper articles. This was done to identify apps described in the press and which might subsequently be searched in either the App Store or Google Play Store via the name of the app in question. InfoMedia was searched in the period 2011–2021 using the search criteria: “app”/”apps” combined with “occupational health”. Twenty-nine articles were found in InfoMedia, referring to ten unique apps. Three new apps were identified through this method. The articles providing additional knowledge about apps identified in the App Store and Google Play Stores became part of the data material.

**Fig. 1:**
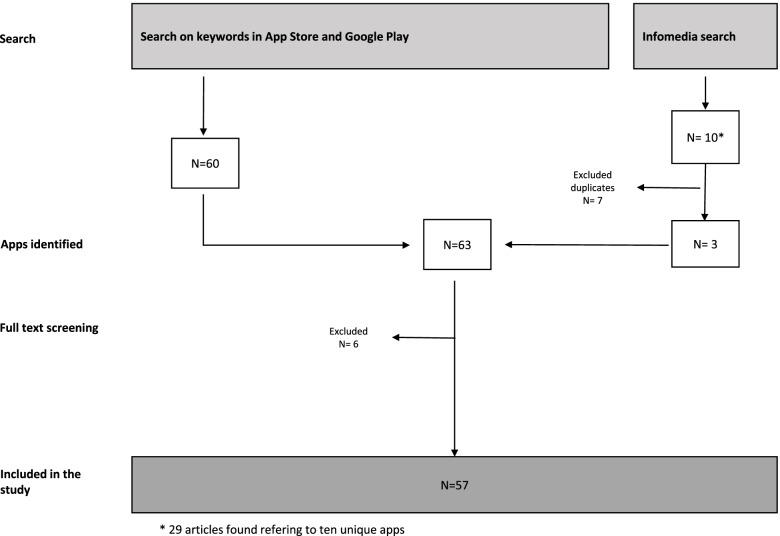
Workflow diagram of app identification and selection process

This process yielded 63 apps for OHS&W. These apps were entered in Nvivo11. Hereafter, we did a second round of exclusion processes following the same criteria as in the first round (mentioned above) but accessing the apps and the description of the apps more thoroughly (full-text screening). In the second exclusion process, six apps were excluded. See Fig. 1 for a diagram of the app identification and selection process.

### Data analyzing processes

We developed a coding system in Nvivo11 to analyze the apps. Codes were made in a dialectical process where two of the authors applied predefined principles (target group, sender of the app, area of occupational health, type of app, reference to research) to the apps, met to discuss them with the third author, made code alterations to analyze the data material better, and applied the new set of codes. This was done in three iterations until a comprehensive categorization was found. 

In the following, we present the final taxonomy we have developed in the study to categorize the apps. We have divided the apps according to which OHS&W area they cover, the target group for the app, the app provider, and the type of app in question (intervention or information/communication). 

### Different fields of occupational health

To create an overview of the field, we categorized the mapped apps according to the type of occupational health area they cover. The apps are coded according to the following categories: Musculoskeletal disorders, psychosocial work environment, work accidents/safety, chemistry, noise, management, rights/legislation, OHS&W coordination (including, e.g., apps for handling workplace assessment or apps that could be used for the safety representatives’ work).

### Sender and recipients

In addition, we have categorized who the developer/owner of the app is (private company, public institution, public/private partnership, cooperative, foundation/non-profit, social partners, industry associations, UN, research institutions) and who the target recipient/audience is (companies, HR-personnel, safety representatives, managers, employees, and occupational health professionals).

### The type of app

In the initial search, we found that OHS&W apps cover a wide range of diverse apps. To operationalize the type of apps, we divided them into two qualitatively different categories: 1) apps that primarily present information (information apps) and 2) apps that aim to create a change in the workplace (intervention apps).

The first category includes apps presenting information and tools for communication, for example, datasheets, information on materials/chemistry, etc., in a digital form. The second category includes apps that introduce a form of intervention in OHS&W, such as prompting workers to answer questionnaires or undergo training. However, the categories are difficult to keep completely separate, as comprehensive and well-accessible information might be a basis for a change in, e.g., work performance and thereby have a derived OHS&W significance. An example is the Danish Emergency Management Agency’s App “Dangerous substances”, an inventory of relevant information on harmful chemical substances. The app contains instructions for the safest possible action in an accident with dangerous substances and the possibility of looking up facts and legislation regarding chemical substances. We thus categorize the app as an information app [1], corresponding to the Danish Emergency Management Agency’s characterization of the app as a reference work; however, the app provides an obvious potential for adapting the work and creating better working environment conditions based on the data provided.

Nevertheless, we have kept the distinction between ‘information apps’ and ‘intervention apps’ as it allowed us to take a closer look at the apps used for OHS&W interventions and examine the degree of documentation for the promised effect. An examination of the effect is not equally relevant for apps that have the format of fact sheets/reference works or apps that make knowledge accessible quickly (contact information, legislation regarding OHS&W, recommended strain in physical work, etc.).

### Assessment of research basis for the effect of the app

**Fig. 2:**
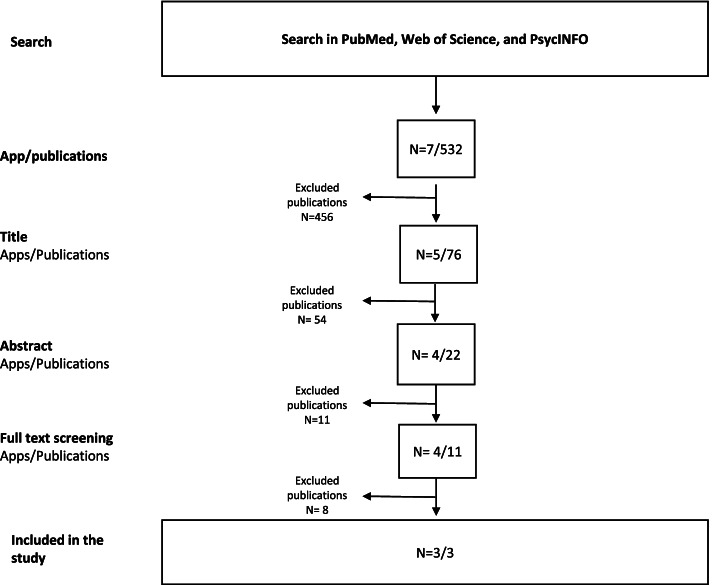
Workflow diagram of literature search and study selection

We assessed how the 37 intervention apps documented the app effects by assessing if they referenced research. We did this using two methods: First, we screened the publicly available data material collected in our app store and InfoMedia searches. This comprised of online information on the app (often the homepage for the app), newspaper articles found in InfoMedia, and the descriptions provided in the App Store and Google Play Store. Second, to ensure that all scientific publications on the specific apps were found, we searched PubMed, Web of Science, and PsycInfo for articles between 2002 and 2021 (see Fig. 2). We searched on the app name plus “app or application” (i.e., “Wysa” + “app”) to identify relevant studies on the identified apps (see Additional file 2). We did three rounds of screening based on the title, abstract and full text of the identified studies counting the number of apps evaluated and the number of publications.

On this basis, we divide the intervention apps into two categories: “not research-based” (apps where we did not find any reference to research in either method) and “research-based” (apps where we find reference(s) to research for all or parts of the mechanisms within the app).

## Results

In total, we found 57 OHS&W apps (see Additional file 1 for the list of apps identified).

**Fig. 3:**
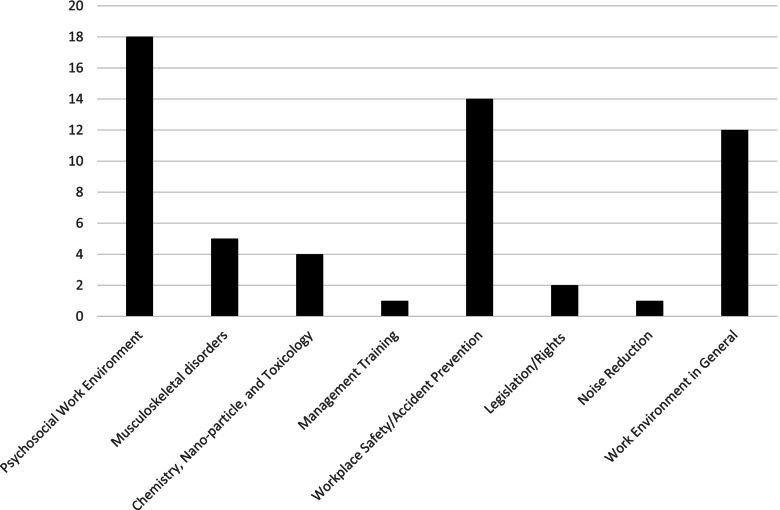
Distribution of apps concerning the area of occupational health

In Fig. 3, the areas targeted by the apps are visualized. Each app is only categorized once based on the app’s main focus to provide an overview of the distribution of apps within work environment areas. An app classified as primarily targeting musculoskeletal disorders might also contain mentions of legislation regarding heavy lifting, but is still classified as an musculoskeletal disorder app as this is its primary focus. The apps were categorized into eight different work environment areas. Most apps were identified in the following three categories; We have classified eighteen of the OHS&W apps as aimed at the psychosocial work environment (such as apps for stress reduction and improved well-being, e.g., Howdy, an app measuring employees’ well-being, with added possibility for counseling if employee score suddenly drops). Fourteen apps were about improving workplace safety (including apps used to document accidents or register events that could lead to accidents, such as Safety Observer). Twelve were classified as apps aimed at ‘OHS&W coordination (e.g., apps for sending out the mandatory workplace risk assessment, such as MusSkema, an app that primarily provides tools for the employee development interviews between the employees and the managers).

Five of the identified apps were aimed at musculoskeletal disorders (apps guiding lifting techniques reducing back pain, such as ErgoArmMeter, a professional inclinometer for measuring and recording arm elevation during work). We classified four apps as relating chemistry and toxicology (e.g., apps about toxic substances in the work environment, such as NanoSafer).

A small number of apps dealt with occupational health legislation/rights [2], apps aimed at OHS management training [1], and apps for noise reduction [1].

**Fig. 4:**
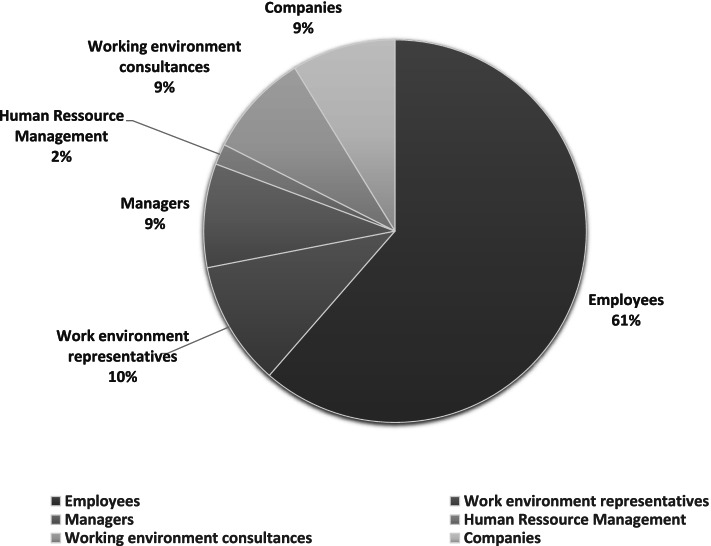
Distribution of apps concerning the primary target audience for the OHS&W apps

More than half (61%) (35 apps) of the identified apps are primarily targeted to employees (see Fig. 4). Nine percent (5 apps) are primarily targeted to managers, 9% (5 apps) are primarily targeted to safety representatives, 10% (6 apps) are primarily targeted to work environment consultants, 9% (5 apps) are primarily targeted to companies, and 2% [1] are primarily targeted to HR departments/consultants.

**Fig. 5:**
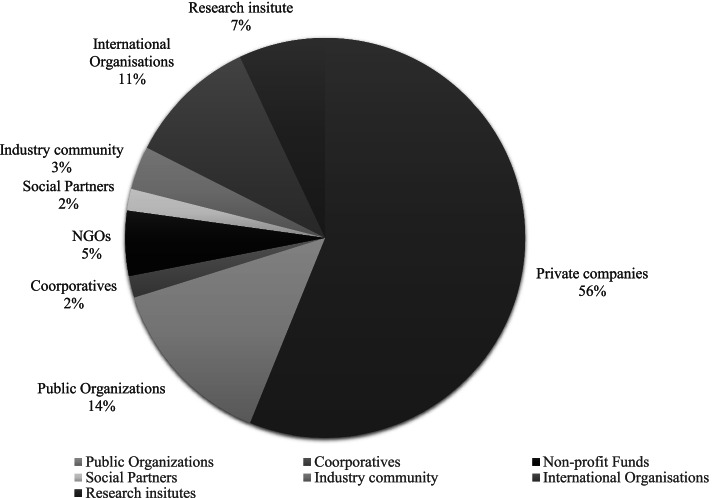
Distribution of apps concerning the sender of the identified apps

Private companies issue 56% [32] of the identified apps (see Fig. 5), 14% [8] are from public organizations, and 11% [6] from international organizations, such as The International Labor Organization. Apps from research institutions cover 7% [4] of the identified apps. Examples include “Safety Observer” (an adaptive safety-screening tool) and “ErgoArmMeter” (an inclinometer for measuring and recording arm elevation during work). The final 12% are distributed between NGOs [3], social partners such as employer organizations [1], industry community [2], and cooperatives [1].

### The type of app

We categorized the apps as either intervention apps or information apps to investigate the extent to which intervention apps’ were based on research and whether the apps’ effects had been evaluated in scientific studies. One app had such sparse information that we could not classify it as an intervention or an information app. Nineteen apps were categorized as information apps. The remaining 37 apps were classified as intervention apps.

### Documentation of effect

We assessed how the 37 intervention apps documented the app’s effects by assessing the degree they referenced research in our collection of online publicly available material and three scientific databases. Table 1 shows the number of apps that referenced research in our data material collected from Google Play, Apple Store, InfoMedia, and online searches distributed in occupational health fields.

**Table 1:** Number of apps referencing research in publicly available data material distributed on work environment fields

Occupational health area	Not research based	Research based
Workplace safety and accidents	9	2
Psychosocial work environment	8	6
Musculoskeletal disorders	3	1
OHS&W coordination	4	1
Noise reduction	-	1
Legislation/rights [No intervention apps]	-	-
Chemistry, Nano-particle, and toxicology	-	1
Apps aimed at leaders	-	1
**Numbers of apps found in the category**	**24**	**13**

In total, 13 of the 37 intervention apps referred to some form of research in their presentation on Google Play, Apple Store, in media articles on InfoMedia, or on affiliated webpages. Within each of the investigated occupational health areas, we found apps that referred to research, with the exception of “legislation/rights,” where there were no apps within the category “intervention” to assess. Most references to research were made in apps within the occupational health area “Psychosocial work environment” with six apps. The occupational health area “Workplace safety and accidents” contained two apps referencing research.

Reference to scientific literature ranged from a single reference to scientific literature substantiating the issues proposedly addressed by the app in question to apps being both built on the basis of scientific findings and undergoing some degree of scientific evaluation of their effect. In general, however, there was too little information available in our online data material to assess the degree and quality of the research they were based on.

Furthermore, we conducted a literature search to get a more accurate picture of whether an app had been scientifically evaluated. We searched three scientific databases (PubMed, PsycINFO, and Web of Science) for studies assessing the effect of the 37 apps. This review resulted in 531 publications, of which three studies assessing three different apps were identified.

Table 2 shows the apps for which we found research articles in scientific databases.

**Table 2:** Number of publications found in scientific databases distributed on apps

Apps	No. of scientific articles found	No. of scientific articles studying app effects
Wysa (psychosocial well-being)	3	1
Noise Exposure (noise reduction)	4	1
ErgoArmMeter (musculoskeletal disorders)	1	1
**Total no. of publications**	**8**	**3**

In the literature search, three apps were identified, which had been evaluated scientifically. In addition, a fourth app (Howdy) was assessed in a scientific study [19], identified through the Howdy homepage. The evaluated apps cover apps aimed at measuring ergonomics (ErgoArmMeter), noise reduction (NoiseExposure), and psychosocial well-being (Wysa and Howdy).

Two of the studies found [25, 26] that evaluated two of the identified apps (ErgoArmMeter and Noise exposure) found the apps in question to be less accurate or have a higher error than the apps they were compared to (ErgoExposure and Sound Level Meter). The two apps performing better than the identified apps were not included in the study. The first one was not included as it was not freely available in App Store or Google Play Store, and the second one was not included as it was not aimed particularly at workplaces. The two apps aiming to improve psychosocial well-being (Wysa and Howdy) showed promising results. However, the studies were not tested in a robust study design, and larger samples are required across more extended periods to validate the initial results.

## Discussion

Our study aimed to identify available OHS&W apps. We identified 57 OHS&W apps targeting a large variety of occupational health issues. The main topics were “OHS&W coordination”, “psychosocial work environment”, and “workplace safety”. Private companies brought more than half of the 57 apps to the market, and the apps were primarily aimed at employees. Of the 57 OHS&W apps identified, 37 were intervention apps aiming to create a change in the workplace, 19 contained information, and one app was described in such sparse details that it was not possible to classify it. We found that 13 of the intervention apps had some reference to research and that four apps had been evaluated in scientific studies. However, two of the identified apps turned out to be less accurate than the apps they were compared to, and two studies showed a positive effect but used suboptimal pilot and quasi-experimental [19, 20] designs instead of randomized controlled trials.

### The proliferation of apps

Of the identified OHS&W apps, most were aimed at psychosocial well-being, second-most were within workplace safety/accident prevention, and third most were within OHS&W coordination. These results are in line with our expectations. We expect methods within psychosocial well-being (mainly surveys), workplace safety/accidents (checklists), and OHS&W coordination (mainly tables and checklists) to be well suited for transfer to an app format aimed at the individual employees. In contrast, more complex work environment interventions might be more challenging to transfer to the app format. We find that a large group of apps is aimed at OHS&W coordination. These apps target safety representatives to support their work.

We find an interesting distinction between this kind of processual support for safety representatives and apps targeting specific challenges in the work environment, e.g., the app “Ladder Safety”, which provides guidance for the correct positioning of ladders. We present the distinction between ‘processual OHS&W apps’ and ‘OHS&W apps targeting specific issues’ as a significant distinction in the market for OHS&W apps. For potential users of OHS&W apps, the first question is whether the need is for a general processual tool to support and digitize processes or an app that will help with a specific occupational health issue.

### Research basis of OHS&W apps

Our results show that approximately a third (13 of 37) of the apps categorized as intervention apps referenced some research in their description.

The range of different methods, study designs, and sources presented as reference to research by app developers should make us cautious not to consider a self-described reference to research as proof of solid study designs examining app effects. In the online presentation of apps, references to research contained widely different things (e.g., everything from mentioning a theory as inspiration for an app’s development to an app based on a validated questionnaire). In summary, we consider it a considerable challenge for occupational health practitioners to assess which OHS&W apps are based on research and the extent to which one can expect that there will be an effect of using the app.

Furthermore, we found only a few [3] scientific evaluations of app effects in our search into three scientific databases for the 37 intervention apps, and one that was known to the authors in advance but did not show up in the search as the app was not mentioned. Two of the studies indicated positive results; however, the studies did not have sufficiently robust study designs to make claims about the apps’ effects. This low degree of research-based evidence resembles findings from reviews in comparable fields where the use of apps is likewise growing rapidly, e.g., mental health technologies and behavioral intervention trials [6, 16, 23]. Our results point toward a lack of scientific studies of OHS&W apps and their effects like these broader fields.

This raises an important point for the scientific community concerned with evaluating app effects [27]. Previous research notes a need for health apps to be better evaluated to ensure their effectiveness and guide consumers [28, 29]. New apps are frequently being released, and current apps often change quickly. Both are factors that speak against the possibility of relying on time-consuming and expensive evaluation designs like RCT to provide research-based evidence on the effects of the apps in a timely manner [6, 30]. In internet-delivered mental health care treatment, where systematic reviews and meta-analyses of RCT studies have been conducted, results have been moderately promising, but most so when coupled with non-online support [31, 32]. We expect these promises and challenges to apply to OHS&W apps as well, added to the challenges of a rapidly changing app market. As robust study designs of app effects are not common presently, and with the possibility that such time-consuming studies will prove to be obsolete as the app market changes rapidly, what is the best way to evaluate OHS&W apps that helps OHS professionals to choose the best app? One way forward is to consider adaptations of RCT designs, either by adjusting the design as apps are upgraded or by employing studies that resemble RCT design as much as possible. Kumar et al. discuss this and provide an overview of evaluation design alternatives to RCT studies for M-health intervention [27]. Another way forward is to employ non-RCT evaluation designs such as quasi-experiments. In this line, research has pointed to evaluating the underlying principles of the app [18, 30] or employing studies that “emphasize usefulness, applicability, and feasibility of new technologies and evaluate them with patients” [29].

### OHS&W apps as practical tools

Our concern is how research can provide information and guidance to occupational health professionals on which apps to implement at workplaces. We want to stress that the scientific approval of OHS&W apps might not be the most important factor for occupational health experts. Many apps will likely have a practical effect, positive or negative, at workplaces as we wait for research to be conducted, or even without it being scientifically evaluated. As with other tools, app use is about finding the right fit for the challenges in consideration of a range of contextual factors [33]. Mohr et al. [34] suggest that digital mental health technologies (internet delivered and apps) are better viewed as technology-enabled services than products. Inadequacy of the previous conceptualization is that digital mental health technologies became considered the primary agent of change. Instead, it is important to evaluate the ecosystem around that technology (such as human support and organizational factors) [29]. We believe this applies to OHS&W apps as well.

Occupational health professionals should not be discouraged from using OHW&S apps altogether. For the 19 apps, we found that primarily contain information, it might not be possible or relevant to assess the effects as they do not aim to make immediate changes at the workplace but are simply an appropriate tool supporting necessary work procedures. Alternatively, apps may not themselves be the subject of a research study, but they may be the tool with which data is collected for work environment research, e.g. [35, 36].

### How apps as technologies might affect OHS&W

In this study, we defined an OHS&W app as “an application accessible through and designed for use on smartphones,” and consider OHS&W apps as specific forms of OHS&W tools addressing OHS&W issues with a particular thematic framing with a specific material (digital) setup. As such, we should be considerate of how apps frame the OHS&W topics they address by way of how they present the OHS&W topic and solutions. The tools employed tend to define the problem they were meant to address [37]. Another perspective is that employee identities are malleable, and apps can become a tool for normative control to regulate employee identity [38]. One potential is that OHS&W apps can individualize occupational health and safety work, as occupational health can potentially become an issue handled between the individual employee and his/her smartphone – rather than between the individual and the organization. As such, OHS&W apps can be seen as part of a broader movement centering on the individual, similarly found in the trend toward “personalized medicine” [39]. It is worth paying attention to this trend. We see benefits within E-health from repeated personal measurements that can be used to follow individuals between treatments [40] or promote personalized medicine [41]. Likewise, the opportunity to create tailor-made individual solutions, e.g., for the individual body (exercise programs for back pain) or the individual well-being (by tracking well-being and providing individual support), provides the potential for benefits that are similar to those in the E-health [41]. However, it may also bring challenges to occupational health. When seeking to improve, e.g., in the psychosocial work environment, organizational interventions are often stressed as the most appropriate and effective [42]. It is also a concern in relation to mental health technology [29]. A risk is that an increasingly individualistic focus on work environment issues will further an individualistic conception of occupational health practices. Ways that OHS&W apps can have an organizational integration is by functioning as bridges between in-person treatment sessions [43] or with a consultancy service of psychologists as an add-on to the app (this is among other places seen in the app Howdy).

### Future research into OHS&W apps

Further research into OHS&W apps’ potential to change conceptions and approaches to occupational health is needed. We hope that occupational health research in the future will contribute to research into the associations between apps and worker health, as well to engage in discussions of how to best evaluate the OHS&W apps with regard to scientific validity and practical feasibility. For now, there are benefits – ease of use, accessibility, real-time measurements—to be gained by occupational health professionals by using OHS&W apps, but major disadvantages – especially the lack of validity of app effects – exist that will likely grow.

## Strengths and limitations

The current study has some limitations. First, limitations in Apple and Google’s interfaces meant that we had no control over what was shown in the searches nor had the opportunity to refine our searches. Additionally, we designed the study to focus on the OHS&W apps available to Danish occupational health professionals. The study provides insight into how far we have come with the scientific validation of OHS&W apps in Denmark; however, the Danish focus might be seen as a limitation.

Second, by focusing on apps publicly available on app stores, our search process has likely missed a number of apps developed internally within large organizations. This is at least our impression based on our knowledge of the Danish field of OHS&W apps.

Third, we anticipate we might have missed interesting apps on the app stores that did not match keywords in our search. The keywords were developed as a filter to allow apps engaging with working life to slip through but keep health apps not engaging with working life themes away. Apps in the borderline areas of working life, like performance apps and sleep apps, were included based on whether or not they used the keywords in our search and hence might not have been included in the study. Such borderline cases are almost impossible to avoid.

Fourth, by designing the bibliographic search as we did, we are limited to studies that clearly state the name of the apps they study. From our search of online material, we were aware of Bech et al.’s study of the app Howdy [19]. Still, as the authors do not mention the app’s name in the publication, we could not identify it in our scientific database search. Likewise, we might have missed other apps due to this limitation.

Despite these limitations, we believe this study provides a needed overview of the apps available to occupational health professionals and the extent to which there is evidence of their effect.

## Conclusion

OHS&W apps cover a wide area and are very diverse. There is no conceptual clarity about what can be categorized as OHS&W. In this study, we have proposed distinguishing between two types of OHS&W apps: those that are essentially digitalization of information and those that intend to make workplace interventions. We assessed the latter category, and found that very few of the apps had been scientifically evaluated. This should be kept in mind when applying apps in workplaces, and future studies may look into scientifically assessing the effect of OHS&W apps. Additionally, we find a need for research to develop a suitable method to evaluate OHS&W apps that is both scientifically valid and practically feasible. Until then, OHS&W apps remain a new and interesting tool for occupational professionals. Finding the right app for a particular problem, and apps that are scientifically evaluated, currently proves to be a substantial challenge.

## Supplementary Information

**Additional file 1.** Overview of included apps.

**Additional file 2.** Search strings.

## Data Availability

All data generated or analysed during this study are included in this published article [and its supplementary information files]. Furthermore, all data generated or analysed as part of this study are publicly available through App Store, Google Play Store, InfoMedia, and the scientific databases searched. The method and the search process are presented in the article making it possible to replicate the study. The identified apps analysed in this study are listed in Additional file 1. The search strings are available in Additional file 2. For questions regarding the study method and data collection, and the data you are welcome to contact the correspondent author.

## References

[1] Khakurel J, Melkas H, Porras J. Tapping into the wearable device revolution in the work environment: a systematic review. Inf Technol People. 2018.

[2] Bregenzer A, Wagner-Hartl V, Jiménez P (2019). Who uses apps in health promotion? A target group analysis of leaders. Health Informatics J.

[3] Whittaker R (2012). Issues in mHealth: Findings From Key Informant Interviews. J Med Internet Res.

[4] Nicholas J, Fogarty AS, Boydell K, Christensen H (2017). The Reviews Are in: A Qualitative Content Analysis of Consumer Perspectives on Apps for Bipolar Disorder. J Med Internet Res.

[5] Dunkl A, Jiménez P (2017). Using smartphone-based applications (apps) in workplace health promotion: the opinion of German and Austrian leaders. J Health informatics journal.

[6] Mohr DC, Burns MN, Schueller SM, Clarke G, Klinkman M (2013). Behavioral Intervention Technologies: Evidence review and recommendations for future research in mental health. Gen Hosp Psychiatry.

[7] European Agency for Safety and Health at Work. Artificial intelligence for worker management: mapping definitions, uses and implications. 2022.

[8] Newlands GJOS (2021). Algorithmic surveillance in the gig economy: The organization of work through Lefebvrian conceived space. Organ Stud.

[9] Salanova M, Llorens S, Cifre E (2013). The dark side of technologies: Technostress among users of information and communication technologies. Int J Psychol.

[10] ILO, Eurofound. Working anytime, anywhere: The effects on the world of work. 2017.

[11] Islam G, Pillet JC, Navazhylava K, Barros M. High-performance connections: Digital holism and communicative capitalism at HappyAppy. Organization. 2021;13505084211057260.

[12] Friis Andersen M, Brinkmann S. Nye perspektiver på stress. Århus: Klim; 2013.

[13] Nielsen K, Noblet A (2018). Organizational interventions for health and well-being.

[14] Nielsen K, Stage M, Abildgaard JS, Brauer CV (2013). Participatory intervention from an organizational perspective: Employees as active agents in creating a healthy work environment.

[15] Wasil AR, Palermo EH, Lorenzo-Luaces L, DeRubeis RJ (2021). Is There an App for That?.

[16] Kertz SJ, MacLaren Kelly J, Stevens KT, Schrock M, Danitz SB (2017). A Review of Free iPhone Applications Designed to Target Anxiety and Worry. J Technol Behav Sci.

[17] Nicholas J, Larsen ME, Proudfoot J, Christensen H (2015). Mobile Apps for Bipolar Disorder: A Systematic Review of Features and Content Quality. J Med Internet Res.

[18] Neary M, Schueller SM (2018). State of the Field of Mental Health Apps. Cogn Behav Pract.

[19] Bech P, Lindberg L, Moeller SB (2018). The Reliable Change Index (RCI) of the WHO-5 in primary prevention of mental disorders. A measurement-based pilot study in positive psychiatry. Nord J Psychiatry.

[20] Bech P, Olsen LR, Kjoller M, Rasmussen NK (2003). Measuring well-being rather than the absence of distress symptoms: a comparison of the SF-36 Mental Health subscale and the WHO-Five well-being scale. Int J Methods Psychiatr Res.

[21] Sandal LF, Bach K, Øverås CK, Svendsen MJ, Dalager T, Jensen JSD, ... & Mork PJ. Effectiveness of app-delivered, tailored self-management support for adults with lower Back pain–related disability: a selfBACK randomized clinical trial. JAMA Intern Med. 2021;181(10):1288-96. 10.1001/jamainternmed.2021.4097 PMC8329791 34338710

[22] Inkster B, Sarda S, Subramanian V (2018). An Empathy-Driven, Conversational Artificial Intelligence Agent (Wysa) for Digital Mental Well-Being: Real-World Data Evaluation Mixed-Methods Study. JMIR mHealth and uHealth.

[23] Wasil AR, Venturo-Conerly KE, Shingleton RM, Weisz JR (2019). A review of popular smartphone apps for depression and anxiety: Assessing the inclusion of evidence-based content. Behav Res Ther.

[24] Naderifar M, Goli H, Ghaljaie F. Snowball sampling: A purposeful method of sampling in qualitative research. Stride Dev Med Educ. 2017;14(3).

[25] McLennon T, Patel S, Behar A (2019). Abdoli-Eramaki MJJoo, hygiene e Evaluation of smartphone sound level meter applications as a reliable tool for noise monitoring. J Occup Environ Hyg.

[26] Öhberg F, Vänn M, Jonzén K, Edström U, Sundström N (2021). Comparison between two mobile applications measuring shoulder elevation angle–A validity and feasibility study. Med Eng Phys.

[27] Kumar S, Nilsen WJ, Abernethy A, Atienza A, Patrick K, Pavel M (2013). Mobile health technology evaluation: the mHealth evidence workshop. Am J Prev Med.

[28] Steigner G, Doarn CR, Schütte M, Matusiewicz D, Thielscher C (2017). Health Applications for Corporate Health Management. Telemed J E Health.

[29] Mohr DC, Weingardt KR, Reddy M, Schueller SM (2017). Three Problems With Current Digital Mental Health Research….and Three Things We Can Do About Them. Psychiatr Serv.

[30] Mohr DC, Schueller SM, Riley WT, Brown CH, Cuijpers P, Duan N (2015). Trials of Intervention Principles: Evaluation Methods for Evolving Behavioral Intervention Technologies. J Med Internet Res.

[31] Richards D, Richardson T (2012). Computer-based psychological treatments for depression: a systematic review and meta-analysis. Clin Psychol Rev.

[32] Kuester A, Niemeyer H, Knaevelsrud C (2016). Internet-based interventions for posttraumatic stress: A meta-analysis of randomized controlled trials. Clin Psychol Rev.

[33] Nielsen K, Randall R (2015). Assessing and addressing the fit of planned interventions to the organizational context.

[34] Mohr DC, Weingardt KR, Reddy M, Schueller SM (2017). Three Problems With Current Digital Mental Health Research and Three Things We Can Do About Them. Psychiatr Serv.

[35] Kirkegaard ML, Kines P, Nielsen HB, Garde AH (2018). Occupational safety across jobs and shifts in emergency departments in Denmark. Safety Sci.

[36] Jensen KA, et al. Performance testing, calibration and implementation of a next generation System-of-systems Risk Governance Framework for nanomaterials – Final technical report Part B. Calibrate. Horizon 2020 grant no. 686239. 2020.

[37] Jöhncke S, Svendsen MN, Whyte SR, Hastrup K (2004). Løsningsmodeller: Sociale teknologier som antropologisk arbejdsfelt. Viden om verden En grundbog i antropologisk analyse.

[38] Alvesson M, Willmott H (2002). Identity Regulation as Organizational Control: Producing the Appropriate Individual. J Manage Stud.

[39] Strecher V (2007). Internet methods for delivering behavioral and health-related interventions (eHealth). Annu Rev Clin Psychol.

[40] Schougaard LMV, Larsen LP, Jessen A, Sidenius P, Dorflinger L, de Thurah A (2016). AmbuFlex: tele-patient-reported outcomes (telePRO) as the basis for follow-up in chronic and malignant diseases. Qual Life Res.

[41] Riggare S. E-patients hold key to the future of healthcare. BMJ. 2018;360. 10.1136/bmj.k846 29483151

[42] Nielsen K, Stage M, Abildgaard JS, Brauer CV, Bauer GF, Jenny GJ (2013). Participatory Intervention from an Organizational Perspective: Employees as Active Agents in Creating a Healthy Work Environment. Salutogenic organizations and change: The concepts behind organizational health intervention research.

[43] Leigh S, Flatt S (2015). App-based psychological interventions: friend or foe?. Evid Based Ment Health.

