# Supplementary material for: A review of smartphone applications designed to improve occupational health, safety, and well-being at workplaces
Source: BMC Public Health. 2022 Aug 10;22:1520. doi: 10.1186/s12889-022-13821-6 (PMC9364491; doi:10.1186/s12889-022-13821-6)
Supplement: Supplementary file 1 — Additional file 1. Overview of included apps. [file 12889_2022_13821_MOESM1_ESM.docx]

**Appendix 1: overview of included apps**

| **Apps** |
| --- |
| Cases\\_AKUT_Præhospitalt Center app |
| Cases\\_ErgoArmMeter |
| Cases\\_P1H |
| Cases\\AMOpartner - vejen til et godt arbejdsmiljø |
| Cases\\AMR |
| Cases\\Arbejdsliv |
| Cases\\BFA Bygge & Anlæg |
| Cases\\Bygergo |
| Cases\\Byggesikkerhed.dk |
| Cases\\ConnectOSH - Participation for Wellbeing |
| Cases\\Coopung arbejdsmiljø |
| Cases\\Daily Diversity in the Workplace |
| Cases\\Det motiverende arbejdsmiljø |
| Cases\\Dit Arbejdsliv |
| Cases\\DoBetter |
| Cases\\Eliminating Child Labour |
| Cases\\Eliminating Forced Labour |
| Cases\\Engage |
| Cases\\Farlige Stoffer |
| Cases\\Godt set |
| Cases\\Howdy |
| Cases\\IA - avigelser i arbejdsmiljø |
| Cases\\iLEAD |
| Cases\\ILO Agriculture Checkpoints |
| Cases\\ILO Ergonomic Checkpoints |
| Cases\\ILO Stress Checkpoints |
| Cases\\Kemi-basen |
| Cases\\KOBOTS |
| Cases\\Ladder Safety |
| Cases\\Leadership for Zen Performer |
| Cases\\Let's master Anti-Racism |
| Cases\\Løftetjek |
| Cases\\MedHelp Manager |
| Cases\\MedHelp Medarbejder |
| Cases\\MS5 (SafeSec) |
| Cases\\Musskema |
| Cases\\NanoSafer |
| Cases\\Noise Exposure |
| Cases\\Office Workout Øvelser |
| Cases\\Quentic |
| Cases\\Risk Assessor Pro |
| Cases\\Rosespillet |
| Cases\\Safety First |
| Cases\\Safety Observer |
| Cases\\SafetyNet EHSQ |
| Cases\\Smash the Office - Stress Fix! |
| Cases\\SOSWEB |
| Cases\\Stresstesten |
| Cases\\Styrpåstofferne |
| Cases\\TeamEffect |
| Cases\\Thrive - Mental Wellbeing |
| Cases\\ToEcho_NemByg |
| Cases\\Wavers |
| Cases\\WebHR |
| Cases\\WOBA |
| Cases\\Worxs |
| Cases\\Wysa - stress, depression and anxiety therapy chatbot |

The URL of Google Play Store used is <https://play.google.com/store>
