# Supplementary material for: A review of smartphone applications designed to improve occupational health, safety, and well-being at workplaces
Source: BMC Public Health. 2022 Aug 10;22:1520. doi: 10.1186/s12889-022-13821-6 (PMC9364491; doi:10.1186/s12889-022-13821-6)
Supplement: Supplementary file 2 — Additional file 2. Search strings. [file 12889_2022_13821_MOESM2_ESM.docx]

**Appendix 2: Search strings**

**PubMed:**

| Search | Query | Items Found |
| --- | --- | --- |
| 1# | “Name of the app” AND **((mobile OR phone OR cellphone OR smartphone OR "web-based" OR "webapp*") OR ("mobile applications"[MeSH])))** | 324 |

**Web of Science**

| Search | Query | Items Found |
| --- | --- | --- |
| 2# | **“Name of the app” AND ((app (All Fields) or apps (All Fields)) OR (KP=mobile application))** | 113 |

**PsycInfo**

| Search | Query | Items Found |
| --- | --- | --- |
| 3# | **("*Name of the app*" AND ((mobile or phone or cellphone or smartphone or "web-based" or "webapp*" or (app or apps))).ti,ab.** | 152 |

**Items Found: 589**

**After removal of duplications: 532**
